# Supplementary material for: Novel Driver Strength Index highlights important cancer genes in TCGA PanCanAtlas patients
Source: PeerJ. 2022 Aug 11;10:e13860. doi: 10.7717/peerj.13860 (PMC9375969; doi:10.7717/peerj.13860)
Supplement: Supplemental Information 2 [file peerj-10-13860-s002.zip › COHORTS/genes plots/cohorts/2021_8_16_14_9_distribution_cohorts_males_LIHC.pdf]

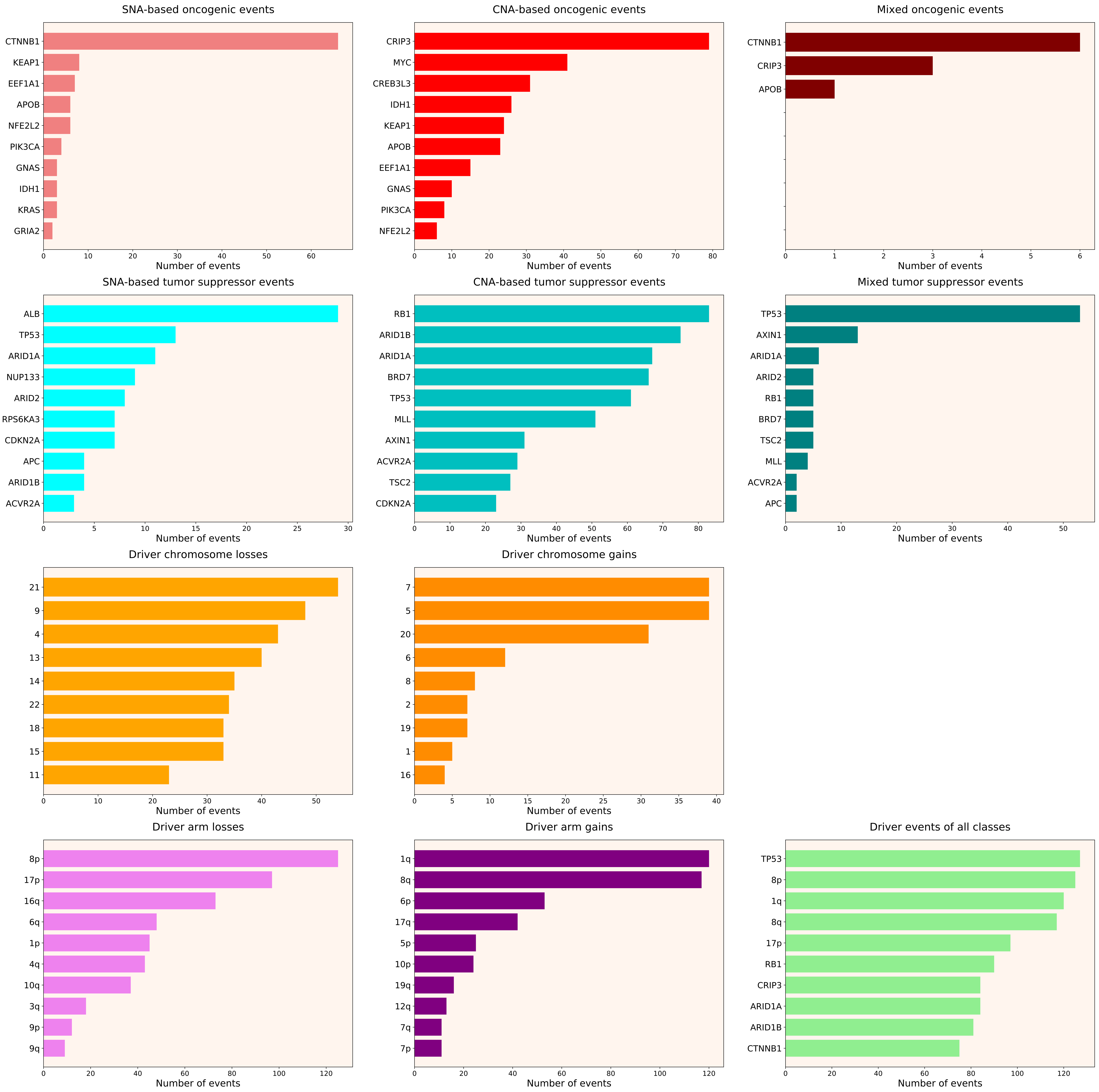
SNA-based oncogenic events
CTNNB1
KEAP1
EEF1A1
APOB
NFE2L2
PIK3CA
GNAS
IDH1
KRAS
GRIA2
Number of events
CNA-based oncogenic events
CRIP3
MYC
CREB3L3
IDH1
KEAP1
APOB
EEF1A1
GNAS
PIK3CA
NFE2L2
Number of events
Mixed oncogenic events
CTNNB1
CRIP3
APOB
Number of events
SNA-based tumor suppressor events
ALB
TP53
ARID1A
NUP133
ARID2
RPS6KA3
CDKN2A
APC
ARID1B
ACVR2A
Number of events
CNA-based tumor suppressor events
RB1
ARID1B
ARID1A
BRD7
TP53
MLL
AXIN1
ACVR2A
TSC2
CDKN2A
Number of events
Mixed tumor suppressor events
TP53
AXIN1
ARID1A
ARID2
RB1
BRD7
TSC2
MLL
ACVR2A
APC
Number of events
Driver chromosome losses
21
9
4
13
14
22
18
15
11
Number of events
Driver chromosome gains
7
5
20
6
8
2
19
1
16
Number of events
Driver arm losses
8p
17p
16q
6q
1p
4q
10q
3q
9p
9q
Number of events
Driver arm gains
1q
8q
6p
17q
5p
10p
19q
12q
7q
7p
Number of events
Driver events of all classes
TP53
8p
1q
8q
17p
RB1
CRIP3
ARID1A
ARID1B
CTNNB1
Number of events
